# Altered Energy Metabolism During Early Optic Nerve Crush Injury: Implications of Warburg-Like Aerobic Glycolysis in Facilitating Retinal Ganglion Cell Survival

**DOI:** 10.1007/s12264-020-00490-x

**Published:** 2020-04-10

**Authors:** Jingyi Zhu, Ping Li, Yuan-Guo Zhou, Jian Ye

**Affiliations:** 1 Department of Ophthalmology, Army Medical Center of the People’s Liberation Army (PLA), Army Medical University, Chongqing, 400042 China; 2 Molecular Biology Center, State Key Laboratory of Trauma, Burn, and Combined Injury, Research Institute of Surgery, Army Medical Center of the PLA, Army Medical University, Chongqing, 400042 China

**Keywords:** Optic nerve crush, ATP, Glycolysis, Oxidative phosphorylation, RGC survival

## Abstract

Neurons, especially axons, are metabolically demanding and energetically vulnerable during injury. However, the exact energy budget alterations that occur early after axon injury and the effects of these changes on neuronal survival remain unknown. Using a classic mouse model of optic nerve-crush injury, we found that traumatized optic nerves and retinas harbor the potential to mobilize two primary energetic machineries, glycolysis and oxidative phosphorylation, to satisfy the robustly increased adenosine triphosphate (ATP) demand. Further exploration of metabolic activation showed that mitochondrial oxidative phosphorylation was amplified over other pathways, which may lead to decreased retinal ganglion cell (RGC) survival despite its supplement to ATP production. Gene set enrichment analysis of a microarray (GSE32309) identified significant activation of oxidative phosphorylation in injured retinas from wild-type mice compared to those from mice with deletion of phosphatase and tensin homolog (PTEN), while PTEN-/- mice had more robust RGC survival. Therefore, we speculated that the oxidation-favoring metabolic pattern after optic nerve-crush injury could be adverse for RGC survival. After redirecting metabolic flux toward glycolysis (magnifying the Warburg effect) using the drug meclizine, we successfully increased RGC survival. Thus, we provide novel insights into a potential bioenergetics-based strategy for neuroprotection.

**Electronic supplementary material:**

The online version of this article (10.1007/s12264-020-00490-x) contains supplementary material, which is available to authorized users.

## Introduction

Neuronal death is a major factor that causes permanent neurological disability after traumatic axonal injury, and the prevention of neuron loss has long been the focus of research on multiple fronts [1, 2]. In these research efforts, energy metabolism is a key factor, since central nervous system (CNS) tissues, particularly axons, are among the most metabolically demanding tissues [3, 4].

Early after CNS trauma, injured axons require extra energy to maintain subsequent energy-intensive processes, such as cytoskeletal structural rearrangements [5, 6]. Energy deficits in axons have been reported to decrease neuronal function and survival [7]. Few attempts have been made to investigate the metabolic patterns of traumatized neurons and their translational significance for neuroprotection [8]. However, our understanding of energetics-based pathological mechanisms in injured neurons is rather limited, hindering the development of bioenergetics-based treatments to address neuronal death [9]. Energy in the form of adenosine triphosphate (ATP) is generated by glucose metabolism in the CNS, predominantly through two main routes, glycolysis and oxidative phosphorylation. Each route has its own merits [10]; therefore, these routes are favored by cells in different states and serve diverse cellular processes. For instance, cancer cells and embryonic retinal cells prefer glycolysis and lactate metabolism even with abundant oxygen – this is also termed aerobic glycolysis or the Warburg effect [11–14]. However, the dependence of traumatized neurons on glycolysis and oxidative phosphorylation remains unclear [9]. A better understanding of the precise adaptation of energetic pathways to CNS trauma may be critical for providing novel insights into neuroprotection.

We therefore set out to investigate altered energy metabolism in injured neurons and determine its potential significance for neuronal survival early after trauma using the mouse optic nerve crush (ONC) model [15, 16].

## Materials and Methods

### Animals

All protocols were approved by the Administration of Affairs Concerning Experimental Animals Guidelines of the Third Military Medical University. Eight- to ten-week-old adult C57BL/6 male mice weighing ~22 g–26 g were purchased from the Jackson Laboratory and housed in the Animal Care Center of the Research Institute of Surgery and Daping Hospital (Third Military Medical University, Chongqing, China). The mice were housed in groups of three under a 12-h light/dark cycle at room temperature (RT) of 22°C and given access to food and water *ad libitum*. All efforts were made to minimize animal suffering.

### Experimental Design and Statistical Analysis

Physiological and biochemical indexes were measured on day 1 after surgery, unless otherwise noted, to evaluate the early-stage response.

### Surgical Procedures

Mice were anesthetized *via* an intraperitoneal (i.p.) injection of pentobarbital sodium (50 mg/kg). The conjunctiva of the left eye was incised, and the orbital muscles were carefully moved aside to avoid damage to blood vessels. The exposed optic nerve was crushed 2 mm distal to the eyeball for 20 s using extra-fine self-closing forceps following previously published methods [16–18]. Proparacaine hydrochloride was applied during surgery and postoperatively as a local anesthetic. A sham operation was performed on the right eye as a self-control. All surgery was performed at 08:00, and tissue was harvested at the same time the next day, unless otherwise noted.

### Energy Metabolism Measurements

#### ATP, ADP, and ADP/ATP Ratio Assays

The mice were sacrificed by cervical dislocation and the retinas and optic nerves were removed immediately. Approximately 6 mm of the optic nerve from the optic head containing the lesion was removed immediately and placed in lysis buffer (Cat. S0026, Beyotime, Chengdu, China) to halt metabolism. The tissue was dissected into small pieces and sonicated as described in a previous study [19]. The supernatant was collected after centrifugation at 12,000 rpm for 10 min at 4°C. The protein concentration was measured using a bicinchoninic acid protein assay kit (Cat. P0012, Beyotime). The supernatant was mixed with the prepared reagent and measured using a firefly luciferase-based ATP assay kit (Cat. S0026, Beyotime), an EnzyLight adenosine diphosphate (ADP) assay kit (Cat. EADP-100, BioAssay Systems, USA), and an EnzyLight™ ADP/ATP Ratio assay kit (Cat. ELDT-100, BioAssay Systems) according to the manufacturers’ instructions using a monochromator microplate reader (SafireII, Tecan, Switzerland). ATP concentrations were calculated from a log–log plot of the standard curve and normalized to the protein concentration.

#### Lactate Assay

Retinas were harvested and homogenized as described above. The lactate level in the supernatant was determined using a commercial colorimetric kit (Cat. AMEKO2677, Lianshuo Biological, China) according to the manufacturer’s protocol.

#### Lactate Dehydrogenase (LDH) Activity

Supernatants of freshly dissected, homogenized optic nerves were obtained as described above. LDH activity in the samples was measured using an LDH assay kit (Cat. A020, Jiancheng, Nanjing, China) following the manufacturer’s protocol.

### Staining Protocol

#### Retinal Whole-Mount Immunofluorescence Assay

Retinal ganglion cell (RGC) survival was determined by counting the number of β-III-tubulin (Tuj1)-positive RGCs in retinal whole-mounts. Tuj1 specifically recognizes neuronal tubulin and is used as an RGC marker [20, 21]. The mice were anesthetized and transcardially perfused with 0.9% saline and 4% paraformaldehyde (PFA), after which the retinas were carefully harvested. Four symmetrical radial cuts were made on each retina. After postfixation in 4% PFA for 1 h at 4°C, the retinas were rinsed three times with phosphate-buffered saline (PBS) and simultaneously permeabilized and blocked in a solution of 3% Triton X-100 in 10% normal goat serum (Cat. ab7481, Abcam, Cambridge, UK) in PBS at room temperature. The samples were incubated with the primary antibody (1:600, Cat. ab78078, Abcam) for 2 days at 4°C. The retinas were washed three times with PBS for 15 min each and then incubated overnight with secondary antibodies [1:400, anti-mouse antibody conjugated with Alexa Fluor 488 (Cat. A-11001, Thermo Fisher Scientific, Waltham, MA, USA)] at 4°C.

#### Frozen Retinal and Optic Nerve Sections

Eyeballs embedded in optimal cutting temperature compound were cut into 10-μm sections along the sagittal axis on a freezing microtome. The middle portions of the optic nerves were cut into serial sections 8 µm thick.

#### Diaminobenzidine Cytochrome Oxidase Histochemistry

Mitochondrial activity was investigated using diaminobenzidine cytochrome oxidase (DAB COX) histochemistry. COX is the terminal enzyme in oxidative phosphorylation and a reliable, well-established marker for measuring mitochondrial ATP generation. Frozen optic nerve sections were placed onto microscope slides and immersed. The DAB COX histochemistry procedure was conducted according to the manufacturer’s protocol (Cat. Lt, R20440, Yuanye Biotechnology Co., Shanghai, China).

Five randomly-selected visual fields from each section proximal to the crush site were subsequently photographed. Brown or tan staining of the cytoplasm and/or membrane indicated positive labeling, and the mean optical density of brown or tan staining was analyzed.

#### LDH Histochemistry

A fresh slide of optic nerve was obtained as described above, and LDH was stained following the manufacturer’s protocol (Cat. G2362, Solarbio, Beijing, China). Blue/purple staining indicated positive labeling. Histochemical staining was automatically categorized as strongly positive, positive, weakly positive, or negative; then the number of positive cells was counted, and the samples were rated by ImageJ IHC Profiler (https://sourceforge.net/projects/ihcprofiler/) as listed in Tables S1 and S2 in the Supplementary materials.

#### Image Acquisition and Analysis

All immunofluorescence images were acquired on an SP8 confocal microscope (Leica, Germany), and immunohistochemistry images were captured using a camera (Dfc290, Leica) attached to an upright microscope (Dm1000, Leica Microsystems, Germany).

To obtain RGC counts in retinal whole-mounts, eight fields from each retinal explant were randomly sampled at central and peripheral locations with progressive eccentricity from the optic nerve head through the midline of the four retinal quadrants at distances of 1.0 and 1.5 mm from the margin of the optic disc. Tuj1-positive RGCs in all fields were counted with ImageJ software (developed by Wayne Rasband, National Institutes of Health, Bethesda, MD; available at http://rsb.info.nih.gov/ij/index.html). The counts were averaged for each explant.

### Calculation of Changes in Glycolysis and Respiration Rates

Three types of unilateral optic nerve crush (ONC) models were adopted; saline, carbonyl cyanide 3-chlorophenylhydrazone (CCCP), or 2-deoxyglucose (2DG) was injected 1 h prior to retinal extraction in each group. Then, ATP levels were measured in the following: the contralateral intact optic nerve (CON), the injured optic nerve (ONC), the contralateral intact optic nerve after 2DG treatment (CON_2DG_), and the injured optic nerve after 2DG treatment (ONC_2DG_). The difference between CON and CON_2DG_ is the energy produced by glycolysis in the intact optic nerve (E_2DG/C_), and the difference between ONC and ONC_2DG_ is the energy produced by glycolysis in the injured optic nerve (E_2DG/O_). The ONC/CON ratio was a fixed cutoff point and the ONC_2DG_/CON_2DG_ and ONC_CCCP_/CON_CCCP_ ratios were compared to determine if the glycolysis and respiration rates increased or decreased. The specific calculations are shown in Fig. 5A.

### Reverse Transcription qPCR (RT-qPCR)

TRIzol reagent was used for RNA extraction according to the manufacturer’s instructions. The total RNA concentrations in the samples were measured using a NanoDrop system (Thermo Scientific). Then, the RNA was reverse-transcribed using a reverse transcription kit (Cat. RR037A, TaKaRa Bio Inc.). Gene expression was quantified using a real-time fluorescence-based quantitative PCR kit (Cat. RR039B, TaKaRa Bio Inc.). Beta-actin was used as an internal control to normalize the RT-qPCR readout.

### Flow Cytometry

#### Thy-1-Positive RGC Counting

A previously reported flow cytometry-based Thy-1-labeling RGC counting method was also applied to assess RGC survival rates. RGCs were isolated using the method described by Chintalapudi [22, 23], with some modifications. Suspensions of retinal cells were generated using enzymatic digestion and subsequently filtered through a sterile 70-µm nylon strainer to obtain single-cell suspensions. Each suspension was centrifuged for 10 min at 200 × g, and the supernatant was discarded. The pellet was re-suspended in PBS/1% FBS, and the cell density was determined. Two microliters of rat anti-mouse Thy-1.2 phycoerythrin (PE) (BD Biosciences, Cat. 553014) antibody were used to stain ~5.0 × 10^6^ cells in a 1000 µL volume for 6 min at RT. The samples were analyzed using an ACEA NovoCyte instrument.

#### Mitochondrial Labeling

Mitochondrial function was evaluated with mitochondrial membrane potential-dependent (Δψm) MitoTracker Deep Red FM staining. The cells were incubated with 100 nmol/L MitoTracker Deep Red FM for 8 min at RT. Deep red fluorescence (excitation at 644 nm, emission at 665 nm) was detected through the allophycocyanin channel.

### Derivation and Analysis of Microarray Data

We used the gene set enrichment analysis (GSEA) computational method to analyze results in the form of pathway rankings that indicated which pathways play important roles in injured wild-type mice compared to PTEN-deficient mice [24, 25]. The GSE32309 gene microarray data were downloaded from the Gene Expression Omnibus database [26] (http://www.ncbi.nlm.nih.gov/geo/), which included three normal mice (GSM800486, GSM800487, and GSM800488) and three mice with PTEN deletion (GSM800489, GSM8004870, and GSM8004871) based on the Mouse Gene 1.0 ST Array from Affymetrix, Inc. (Santa Clara, CA, USA) [27]. The original CEL files were converted into expression measures and normalized using the affy package in the R language (http://www.bioconductor.org/packages/3.0/bioc/) [28]. Then, the data were analyzed with GSEA-3.0 software with the default parameters, except for the permutation parameter selection, which was set to “geneset” instead of “phenotype” [25]. The enrichment score, normalized enrichment score, *P* value, and false discovery rate Q value were obtained from the GSEA output reports, and these were then used to rank the gene sets.

### Drug Administration and Effectiveness

Doses of 500 mg/kg 2DG (D8375, Sigma-Aldrich), 4 mg/kg CCCP (C2759, Sigma-Aldrich), 100 mg/kg meclizine (B1786, Apexbio), and 50 mg/kg or 5 µmol/kg ATP (PV3227, Thermo Fisher) were injected intraperitoneally.

We injected 500 mg/kg 2DG or 4 mg/kg CCCP immediately after surgery to investigate the numbers of surviving RGCs after inhibition of glycolysis and oxidative phosphorylation. For the analysis of glucose metabolism (Fig. 5), mice were injected with the same dose of 2DG (500 mg/kg)/iodoacetic acid (60 mg/kg)/oligomycin (0.5 mg/kg)/CCCP (4 mg/kg) 1 h prior to euthanasia.

### Statistical Methods

The experimenter was blinded to the treatment of the mice when collecting and analyzing the data. The data are presented as the mean ± SD, and the error bars indicate the 95% confidence intervals. Paired* t*-tests were applied to compare eyes from the same animal or the optic nerve and retina from the same eye, and independent-samples* t*-tests were used for unrelated samples. Multiple comparisons were performed using one-way analysis of variance (ANOVA) followed by Tukey’s or Dunnett’s *post hoc* test, or two-way ANOVA followed by Sidak’s *post hoc* test. Correlations were analyzed using the Pearson correlation test as appropriate. A* P* value <0.05 indicated statistical significance.

Statistical analyses were performed using Sigma Plot version 13.0 and PRISM version 6.0 (GraphPad Software). Graphics were plotted in PRISM version 6.0 and Adobe Illustrator [29, 30].

## Results

### ONC Triggers Metabolic Activation in Injured Optic Nerves and Retinas

**Fig. 1:**
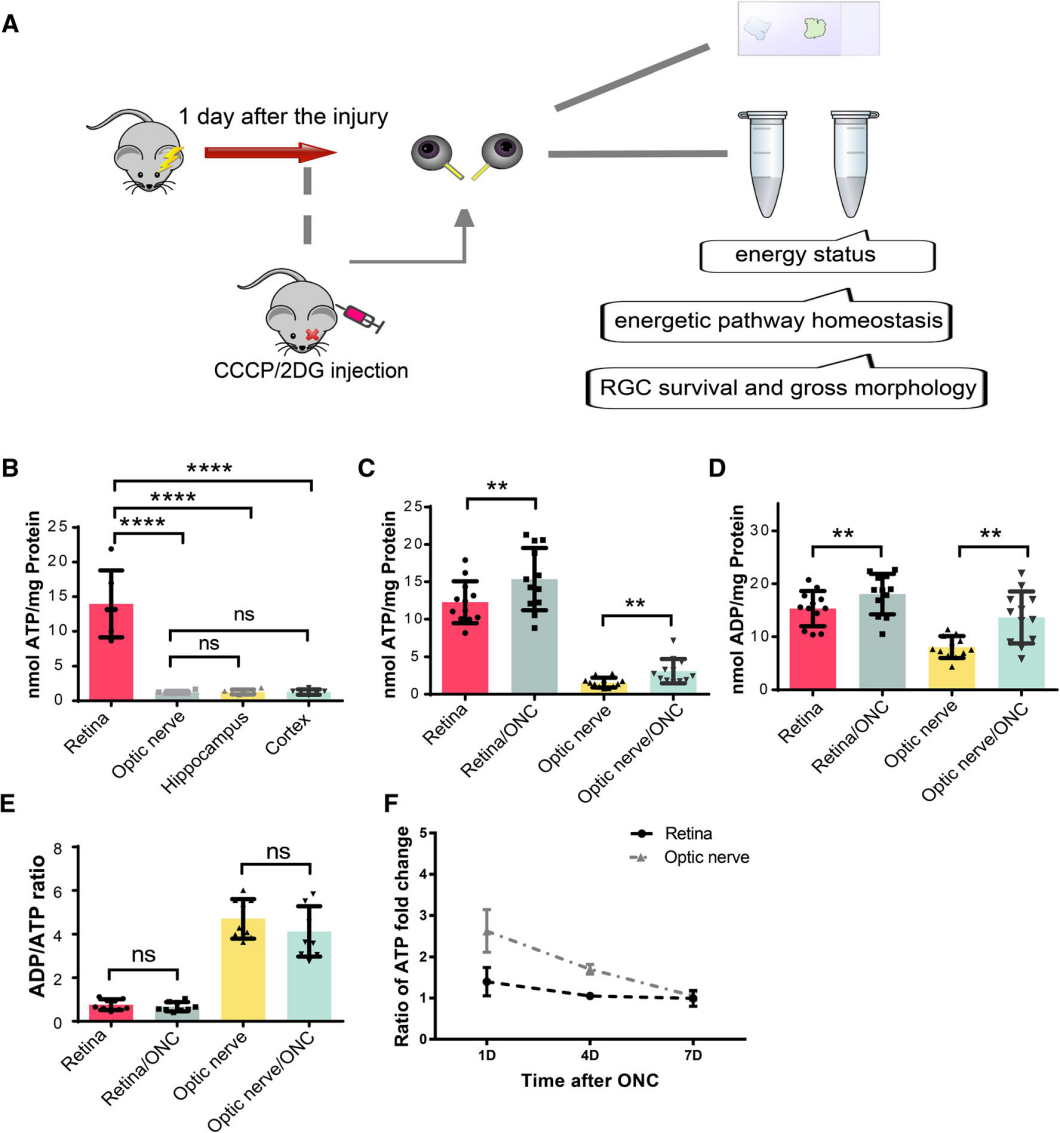
Optic nerve crush (ONC)-stimulated energy metabolism in the retina and optic nerve. **A** Schematic of the experimental design. The main outcome measurements included the energy status (ATP levels, ADP levels, and the ADP/ATP ratio), energetic pathway homeostasis (glycolysis and oxidative phosphorylation activity and relative proportions), and retinal ganglion cell (RGC) status (numbers of surviving cells and gross morphology). **B** Basal ATP levels in the retina, optic nerve, hippocampus, and cortex. **C**, **D** ATP and ADP levels in retina and optic nerve 1 day after ONC. **E** The dynamic balance of energy metabolism presented as the ADP/ATP ratio. **F** ATP concentration-time curves for retinas and optic nerves 1, 4, and 7 days after ONC. **P < 0.01, ****P < 0.0001; ns, not significant.

In this study, we used the ONC model to investigate the role of the energy budget in the pathology of acute traumatic axon injury (Fig. 1A). ONC is a classic animal model of neurodegeneration, with early axon loss and cell death 1 day after injury [31, 32]. The visual system ranks among the most energy-demanding systems in the brain [33]. Consistently, our results showed that the retina had a much higher ATP content than the cortex and hippocampus in mature C57BL/6 mice at baseline (Fig. 1B).

We first measured the energy levels of retinas and corresponding optic nerves and found that the ATP levels were moderately increased after ONC (Fig. 1C). Their ADP levels also increased (Fig. 1D), supporting the hypothesis that neurons rapidly undergo a series of energy-expensive processes in response to injury [5]. The ATP and ADP increases were smaller in retinas than in optic nerves, probably because the nerves were directly traumatized, while only a small portion of the retinal cells (RGCs) was affected. However, the ADP/ATP ratio, a critical parameter of energy consumption and balance [34], showed no decrease, indicating that the relative energy equilibrium was maintained through robust energy consumption and generation (Fig. 1E).

We confirmed the stimulation of energy metabolism in the ONC model. Notably, when monitoring ATP concentrations for a longer period (3 and 7 days after injury) (Fig. 1F), this increase was not apparent in the retina but still occurred in the optic nerve after 3 days. Subsequently, this trend disappeared in both locations 7 days after injury.

**Fig. 2:**
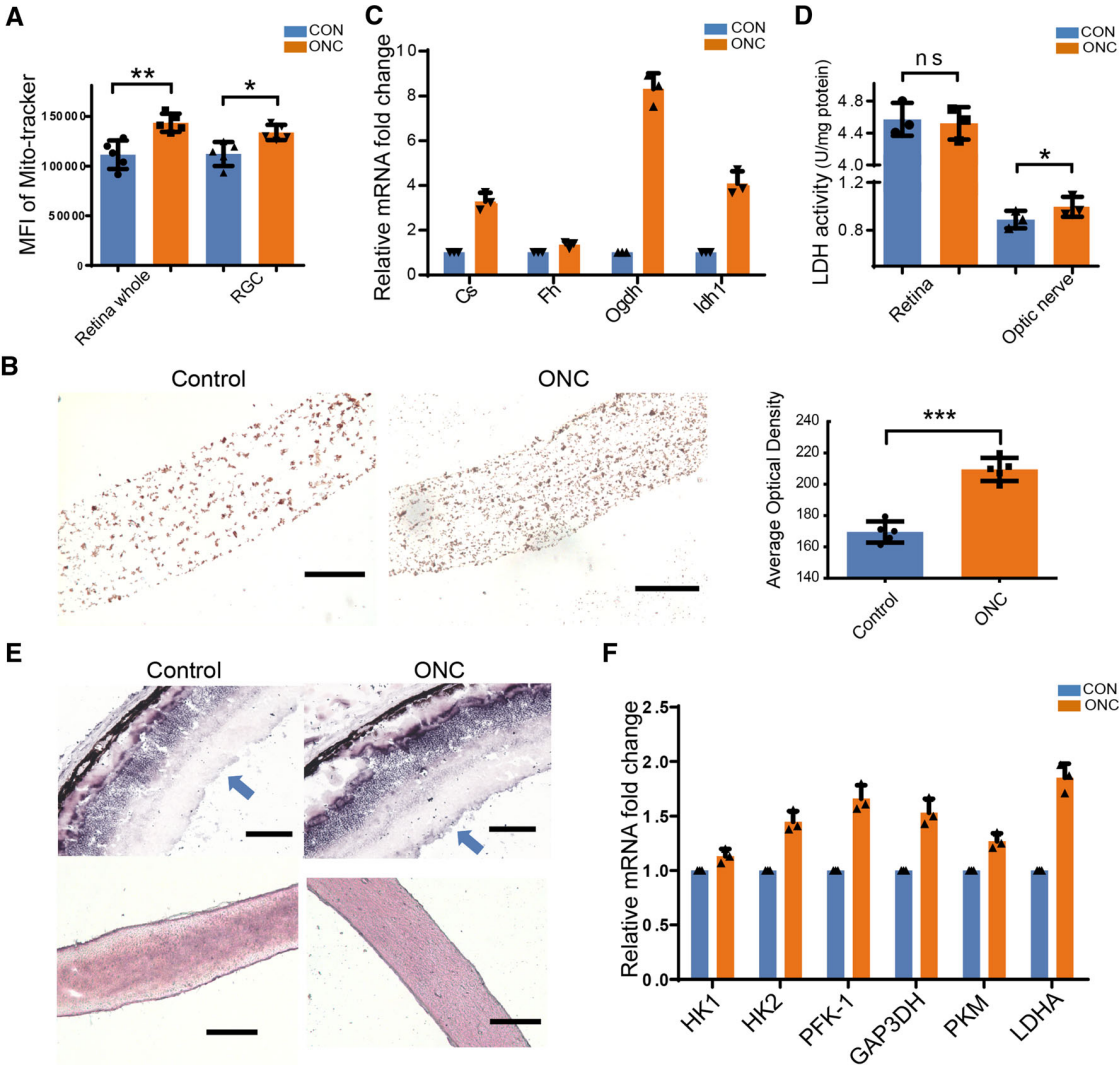
Changes in glycolysis and mitochondrial oxidative phosphorylation after ONC. **A** Statistical analysis of the mean fluorescence intensity of MitoTracker staining in retinal cells and RGCs. **B** Representative images and quantitative analysis of diaminobenzidine staining of cytochrome oxidase in injured and sham-operated optic nerves. **C** mRNA levels of tricarboxylic acid cycle enzymes in crushed optic nerves after ONC. **D** Quantitative analysis of lactate dehydrogenase (LDH) activity in the optic nerve and whole retina after ONC. **E** Representative images of LDH activity (blue/purple staining) in the retina (upper) and the optic nerve (lower) after injury [note intense and disseminated staining in the RGC layer (blue arrows); qualitative analysis in Table S1]. **F** mRNA levels of glycolytic enzymes in optic nerves after ONC. Scale bars in B and E, 100 μm.

We further evaluated the activity of the two main glucose metabolism pathways in the visual system, glycolysis and mitochondrial oxidative phosphorylation. To assess mitochondrial activity in RGCs, we used a previously-reported flow cytometry-based assay. Isolated retinal cells were co-stained with MitoTracker Deep Red, a Δψm-dependent dye that binds to metabolically active mitochondria, and Thy-1.2, an RGC-specific marker. The mean fluorescence intensity of MitoTracker Deep Red staining in Thy-1.2-positive cells indicated that the number of active mitochondria in RGCs increased after ONC (Fig. 2A). We then evaluated mitochondrial COX activity in the optic nerve using DAB staining. A substantial increase in mitochondrial staining was observed in the injured nerve (Fig. 2B). Moreover, the mRNA levels of tricarboxylic acid cycle enzymes (citrate synthase, α-ketoglutarate dehydrogenase, fumarate hydratase, and isocitrate dehydrogenase 1) were increased in the nerve (Fig. 2C). Based on these results, mitochondrial oxidative phosphorylation is enhanced in the ONC model.

The level of glycolysis is indicated by the expression and activity of the glycolytic enzyme LDH, a key enzyme in anaerobic respiration that catalyzes the final step of glycolysis by converting pyruvate to lactate. Quantitative analyses of LDH activity in tissue homogenates revealed a slight increase in injured optic nerve, but this activity remained basically unchanged in the whole retina (Fig. 2D). Qualitative analyses of LDH histochemistry also showed a higher level of staining in injured optic nerve (Fig. 2E and Table S1). The ganglion cell layer of the retina also displayed intense LDH staining in the ONC group, but no significant changes were evident in the rest of the retina (Fig. 2E). Thus, the LDH increase in RGCs may be masked by the remaining part of the retina, as shown in Fig. 2D. qRT-PCR revealed increased expression of the glycolysis enzymes hexokinase 1 and 2, phosphofructokinase-1, glyceraldehyde 3-phosphate dehydrogenase, pyruvate kinase, and lactate dehydrogenase A in optic nerve following ONC (Fig. 2F). Together, these findings indicate a trend of increased glycolysis in injured optic nerve.

### Neither Systemic Increases in Internal Energy Levels nor Supplementation with Exogenous ATP Improves RGC Survival

**Fig. 3:**
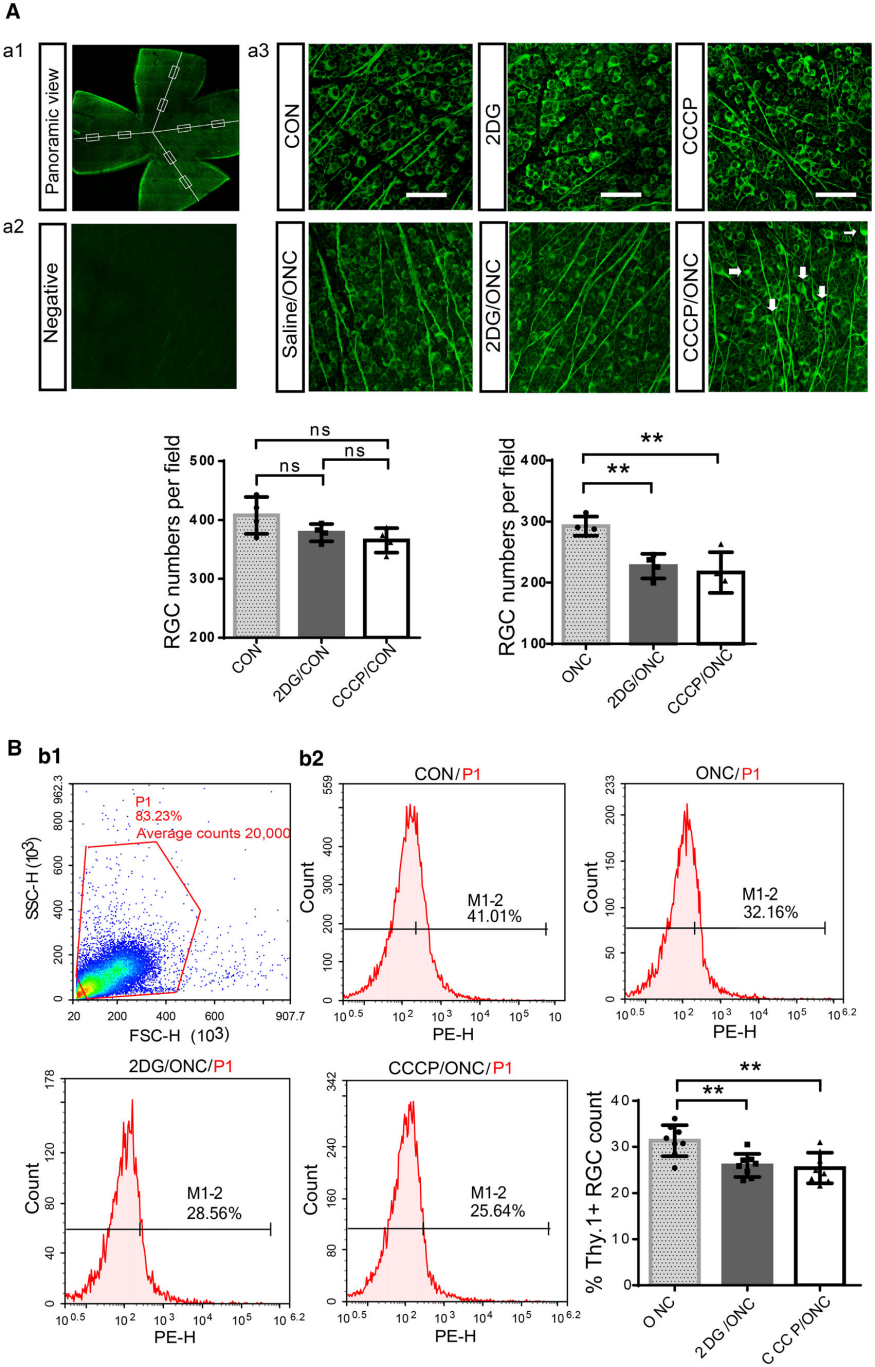
Blocking the ATP supply by inhibiting glycolysis or oxidative phosphorylation increases the retinal damage. **A** Images of flat-mounted retinas stained with Tuj1; eight fields from each retinal explant (boxes) were analyzed. **a1** Representative example of RGC layers in a whole-mount retina stained for Tuj1 (green). **a2** Representative image of the negative control, from which the primary antibody (Tuj1) was omitted. **a3** Representative confocal images from the central regions of retinas in each group. The ONC group with carbonyl cyanide 3-chlorophenylhydrazone (CCCP) injection displayed swollen somata with tubulin accumulation (arrows). Scale bars, 200 μm. **B** Flow cytometry-based sorting strategy for Thy-1.2-positive cells. **b1** An initial gated population (P1) was plotted to discard clumped cells or aggregates and obtain a single-cell population. **b2** Post-sorting analysis of the percentage of Thy-1.2-positive RGCs (M1-2) in each group (note greater RGC death in the 2DG and CCCP groups 1 day after ONC).

What is the biological significance of this energetic activation? Since neurons are metabolically demanding and energy-dependent, we suspected that this post-trauma metabolic response was essential for RGCs to avoid energy deficits and survive. To test this hypothesis, we intraperitoneally injected mice with 2DG, an inhibitor of the glycolytic enzyme hexokinase, and CCCP, a protonophore (H^+^ ionophore) and uncoupler of mitochondrial oxidative phosphorylation, immediately after surgery to restrict glycolysis or oxidative phosphorylation, respectively. The numbers of surviving RGCs were counted in Tuj1-labeled retina whole-mount preparations and using the flow cytometry-based Thy-1.2-labeling RGC sorting method. CCCP and 2DG significantly reduced RGC survival in the ONC model at 1 day; however, there were no significant effects on the intact retina (Fig. 3A, B).

**Fig. 4:**
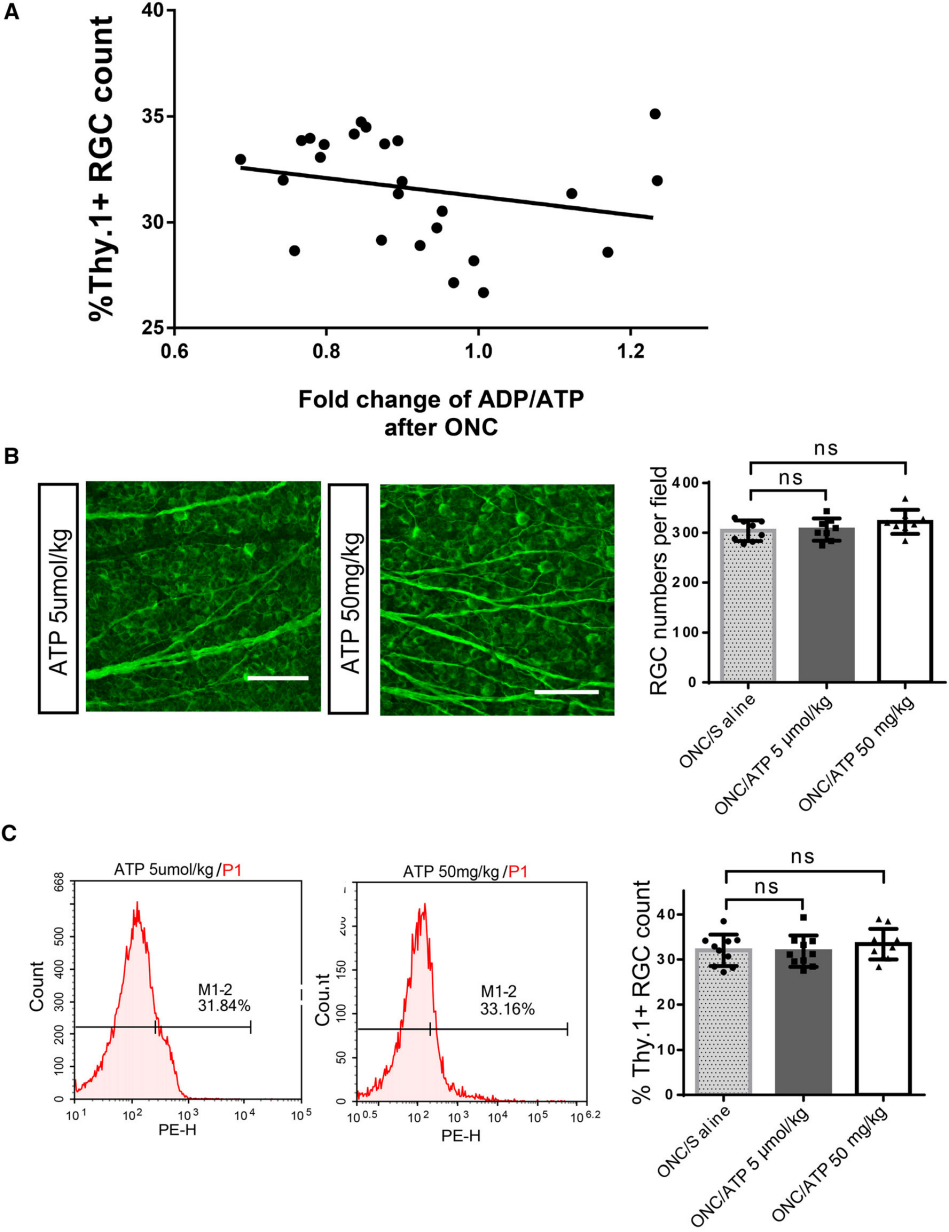
Neither greater endogenous energy reserves nor exogenous ATP supplementation results in reduced RGC loss. **A** Pearson’s correlation analysis revealed no significant correlation between the numbers of Thy-1.2-positive RGCs and ATP reserves (*n* = 25; *r* = -0.2507; *P* = 0.2268). The line represents the linear regression equation of the data (Y = –4.337*X + 35.55, *R*^2^ = 0.06283, not significant),* r*, correlation coefficient. *R*^2^ is calculated from the multivariate linear regression. **B** Neither 5 µmol/kg nor 50 mg/kg ATP injection had an effect on RGC survival after ONC (scale bars, 200 μm). **C** Percentages of Thy-1.2-positive RGCs with and without ATP injection.

Since blocking intense energy production is detrimental to RGC survival, does a larger energy reserve help protect against RGC loss? In other words, does a mouse with greater internal energy levels after injury have an advantage in terms of RGC survival? To determine the inherent energy potential of each mouse after injury, the ADP/ATP ratios of the injured and intact optic nerves from the same mouse were calculated. Pearson correlation analyses and regression analyses were used to examine the relationship between RGC survival and the fold change in the ADP/ATP ratio (Fig. 4A). Surprisingly, no significant correlation was found (*n* = 25; *r* = –0.2507; *P* = 0.2268). Therefore, mice with a more energetically active optic nerve after injury have no advantage in terms of reduced RGC death.

Energy supply tends to promote neuronal survival following CNS injury [35, 36]. Given that more internal energy failed to produce more surviving RGCs, we questioned whether supplying extrinsic ATP would confer any benefit for RGC survival. ATP was injected at a relatively high dose (50 mg/kg) [37, 38] and a low dose (5 µmol/kg; ~2.865 mg/kg) [39]. Intraperitoneal injections did not have a significant effect on RGC survival in either injured or intact eyes (Fig. 4B, C).

### Energetic Reliance Shifts to Mitochondrial Respiration in Injured Optic Nerve

**Fig. 5:**
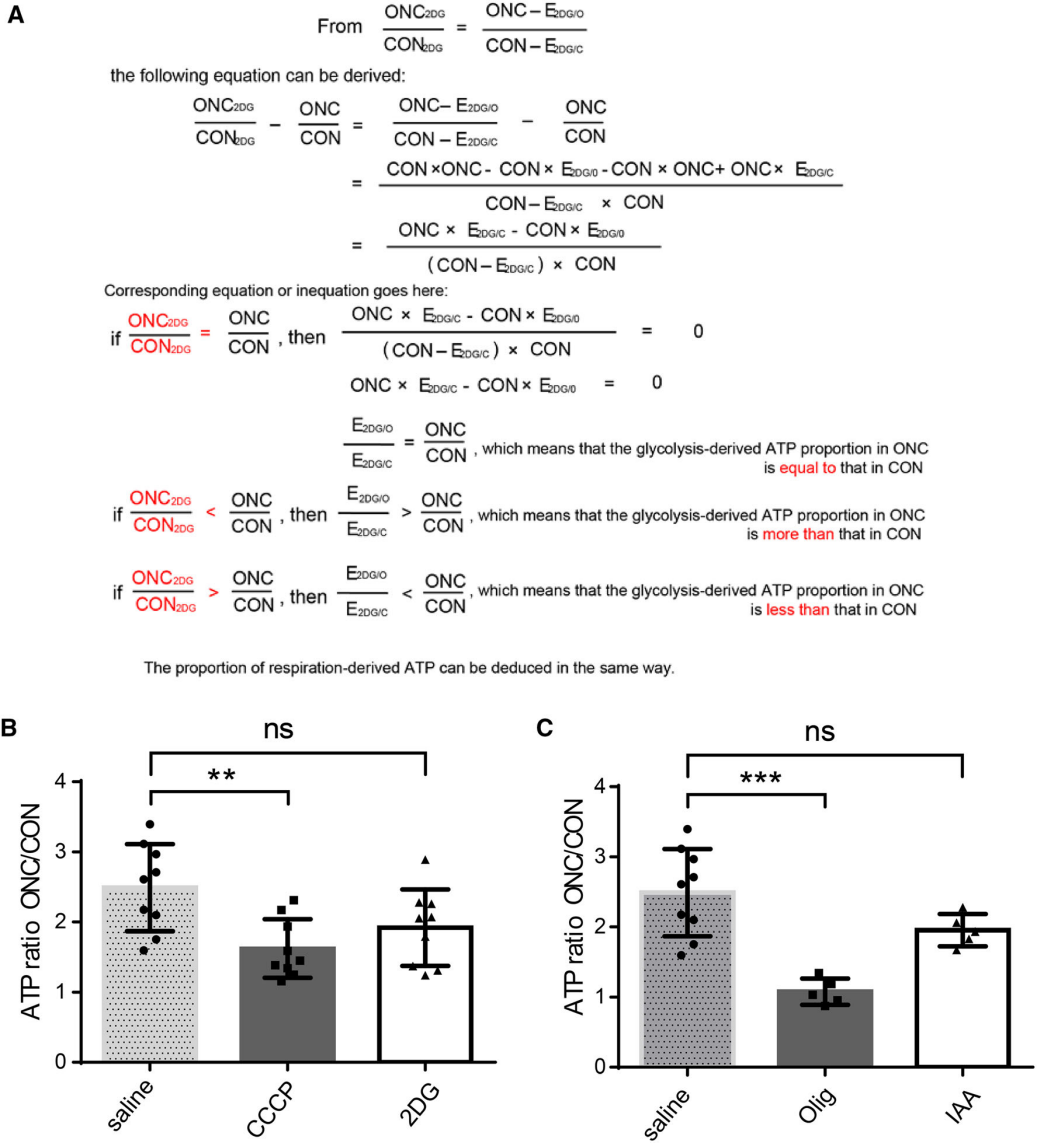
Energy metabolism shifts to oxidative phosphorylation in injured optic nerve. **A** Derivation of the formula for measuring the changes in each pathway and their contributions to energy production. The algorithm establishes ONC_2DG_/CON_2DG_ as an index for evaluating an altered ratio of glycolysis and respiration. **B**, **C** Calculated ATP ratios from injured/intact optic nerves. ONC_CCCP_/CON_CCCP_ and ONC_oligomycin_/CON_oligomycin_ were decreased in traumatized optic nerves (note that the relative contribution of oxidative phosphorylation to the energy supply increased following ONC and the proportion of glycolysis showed no significant change)

Following our observation that both glycolysis and mitochondrial oxidative phosphorylation are upregulated after ONC, we questioned how strongly each pathway became elevated and contributed to ATP production. We established three groups of unilateral ONC models and measured the ATP content in the corresponding optic nerves (the meaning of each value is explained in the Methods). CCCP and 2DG were injected to inhibit respiration and glycolysis, respectively, into ONC mice, while saline was injected into control mice. Both crushed and sham-operated optic nerves were extracted 1 h after the injection, and the ATP levels were measured. According to the formula shown in Fig. 5A, the ATP ONC/CON ratios were set as fixed cutoff points to differentiate changes in the proportions of the different pathways after injury, and the ONC_2DG_/CON_2DG_ and ONC_CCCP_/CON_CCCP_ ratios were compared to the ONC/CON value to determine if the rates of glycolysis and respiration increased or decreased. The data showed that the ONC/CON value after the CCCP injection (ONC_CCCP_/CON_CCCP_) was greater than the control ONC/CON value, while no significant difference was found between ONC/CON and ONC_2DG_/CON_2DG_ (Fig. 5B). These calculations suggested a greater proportion of oxidative phosphorylation in the injured optic nerve than in the intact nerve. For further validation, additional glycolysis and oxidative phosphorylation inhibitors were applied – iodoacetic acid was used to inactivate glyceraldehyde-3-P dehydrogenase, and oligomycin was used to block membrane-bound mitochondrial ATP synthase and proton channels – and they produced similar results (Fig. 5C). Thus, metabolic activation along with a metabolic shift toward respiration occurs in traumatized optic nerves.

The disproportionate increase in respiration caught our attention, raising the question of its biological significance. Is it beneficial or deleterious for RGC preservation? Experiments with PTEN deletion mice helped to provide answers to these questions. PTEN deletion is well established as a general factor controlling neuronal growth, and it has frequently been shown to preserve neurons in the context of CNS injury [40]. Downregulation of PTEN has been shown to increase RGC survival in an ONC model [20, 41]. PTEN is also a metabolic regulator that can suppress glycolysis and cause an energetic shift toward mitochondrial respiration when activated [42, 43]. Based on this evidence, we investigated whether respiration-biased metabolism contributes to the limited neuron survival we found and whether PTEN deletion restricts respiration, thus protecting RGCs.

**Fig. 6:**
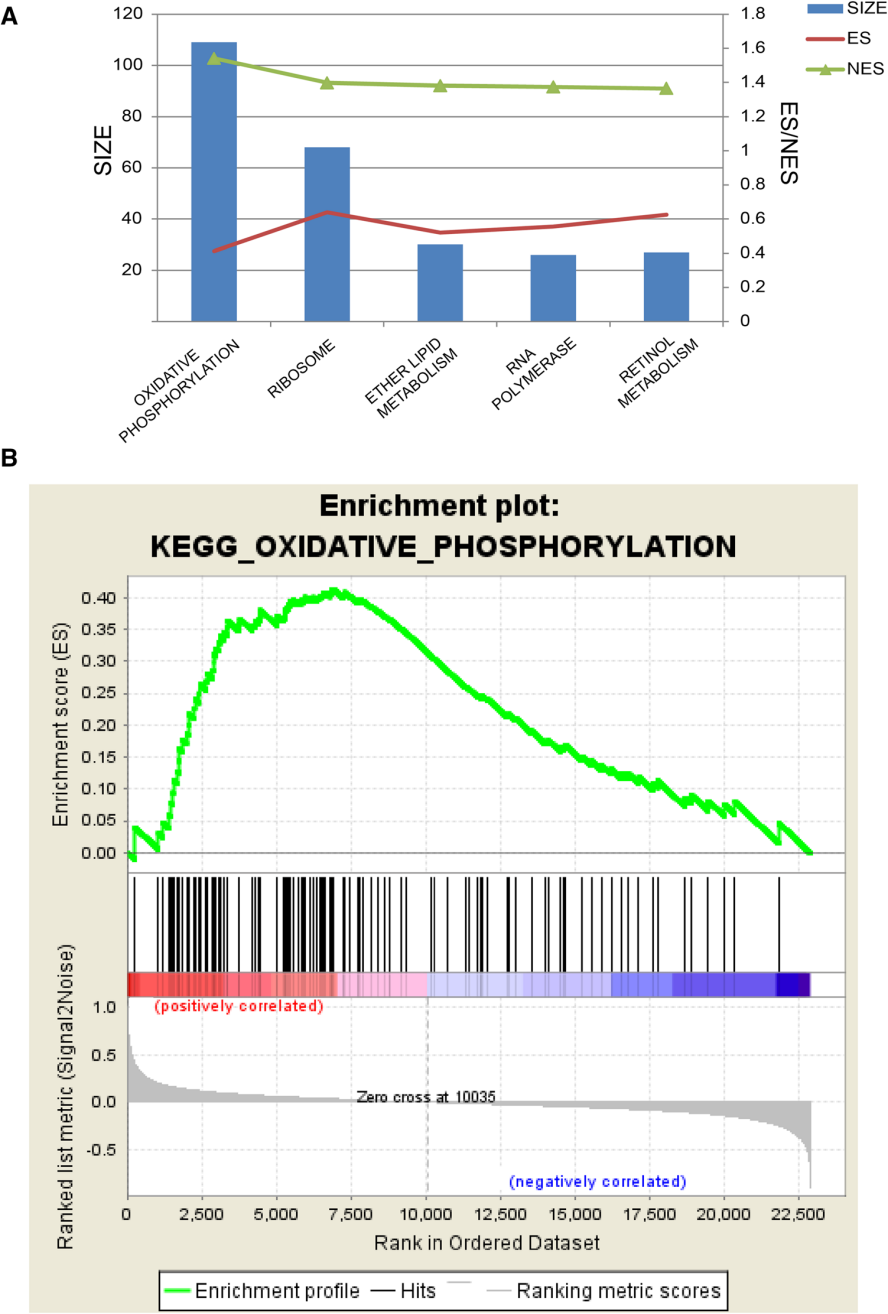
Gene set enrichment analysis demonstrating enriched pathways in injured wild-type *versus* PTEN-deleted retinas. **A** The top five pathways enriched in injured wild-type mice compared to PTEN-deleted mice, with oxidative phosphorylation ranked first. Bar plot, SIZE of the top 5 enriched gene sets; red, enrichment score; green, normalized enrichment score. (ES, the degree to which a gene set is overrepresented at the top or bottom of a ranked list of genes; NES, differences in gene set size and correlations between gene sets and the expression dataset; SIZE, number of genes in the gene set after filtering out those not in the expression dataset). **B** Enrichment plot of the KEGG_OXIDATIVE_PHOSPHORYLATION pathway (note that significant enrichment of “oxidative phosphorylation” genes was found in injured retinal cells from wild-type mice compared with those from PTEN deletion mice).

The GSEA results from a microarray (GSE32309) of PTEN-deficient and wild-type mice subjected to ONC supported our hypothesis. The data revealed that the top five pathways enriched in wild-type mice were KEGG_OXIDATIVE_PHOSPHORYLATION, KEGG_RIBOSOME, KEGG_ETHER_LIPID_METABOLISM, KEGG_RNA_POLYMERASE, and KEGG_RETINOL_METABOLISM (Fig. 6A). Although oxidative phosphorylation was ranked first in the wild-type group, it was not enriched in the PTEN-deficient group (Fig. 6B). Thus, a high level of oxidative phosphorylation is a prominent and potentially adverse process occurring in retinal neurons after optic nerve injury.

### Redirecting Metabolism toward Glycolysis (Warburg Effect) Protects against RGC Loss

**Fig. 7:**
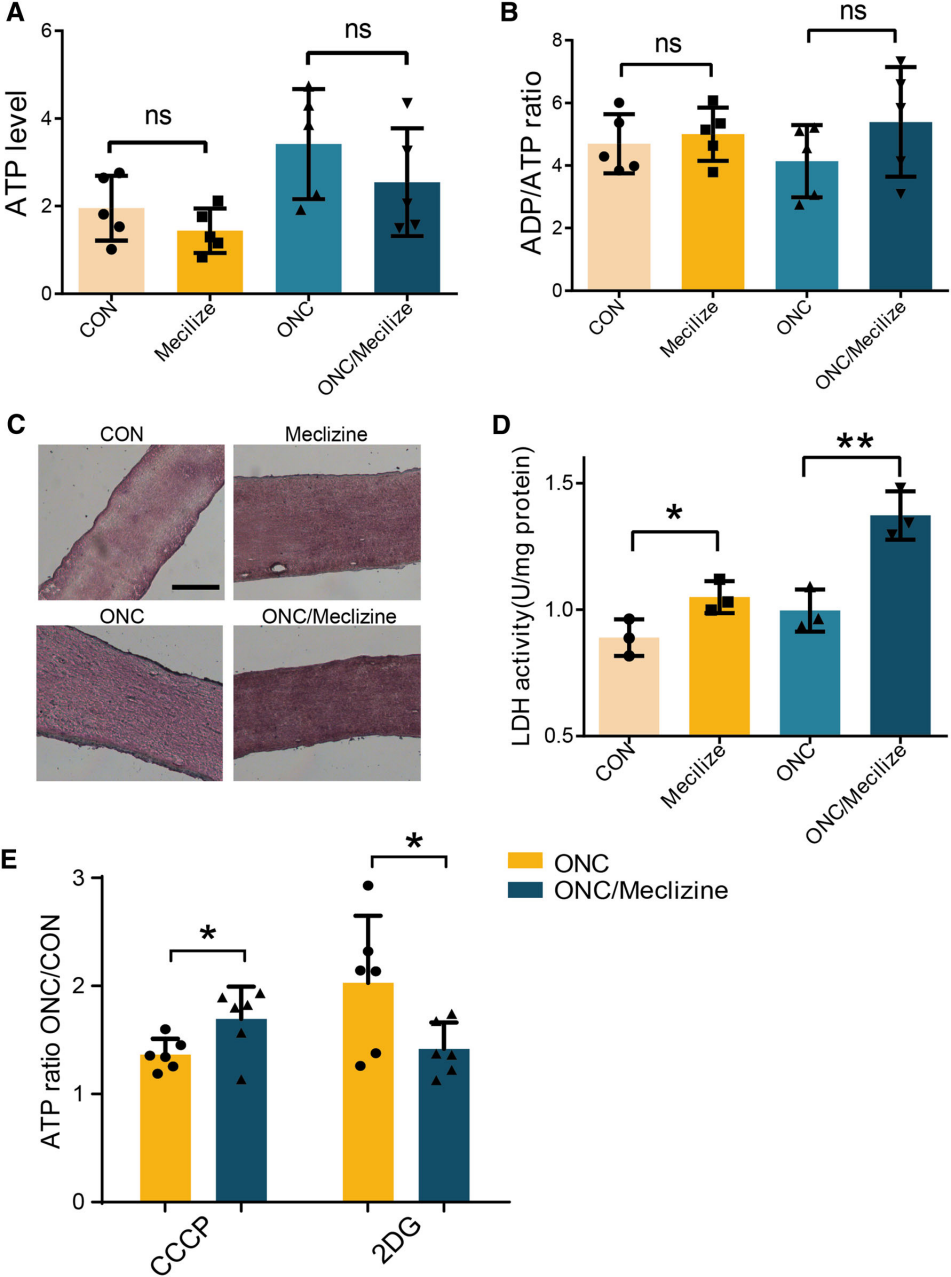
Meclizine redirects metabolic flux toward glycolysis. **A**, **B** Pretreatment with meclizine did not significantly change ATP levels (**A**) or ADP/ATP ratios (**B**). **C** Representative images of LDH staining (note the stronger staining in the meclizine group than in the control group, and in the meclizine pretreatment ONC group than in the ONC group; histochemical ratings in Table S2). Scale bar, 100 μm. **D** LDH activity in the meclizine treatment and the ONC/meclizine groups. **E** Effects of pretreatment with meclizine on ONC_CCCP_/CON_CCCP_ and ONC_2DG_/CON_2DG_ in injured optic nerves.

If the above hypothesis is correct, then restricting oxidative phosphorylation should benefit RGC survival in the ONC model. We tried to increase the activity of another primary energy source, i.e., glycolysis over respiration, similar to the “Warburg effect” in tumors and PTEN-deficient mice, to avoid oxidative phosphorylation overload and simultaneously maintain ATP production. We performed a literature search and identified a drug that shifts energy dependence to glycolysis. Paul S Brookes and Vamsi K Mootha examined > 3,500 small molecules and discovered that the clinically-used drug meclizine attenuates mitochondrial respiration without reducing mitochondrial biogenesis or viability and augments glycolysis in various models [44, 45]. We intraperitoneally injected 100 mg/kg meclizine 17 h and 3 h before ONC, as suggested in previous reports [44, 45], and measured the metabolic effects. Although total ATP levels and the ADP/ATP ratio were not significantly altered in injured optic nerves (Fig. 7A, B), LDH staining and activity were markedly increased in meclizine-treated optic nerves (Fig. 7C, D). The contribution of glycolysis to the energy output increased, while the contribution of mitochondrial respiration decreased (Fig. 7E). Thus, meclizine was able to shift the metabolic dependence toward glycolysis rather than mitochondrial respiration.

**Fig. 8:**
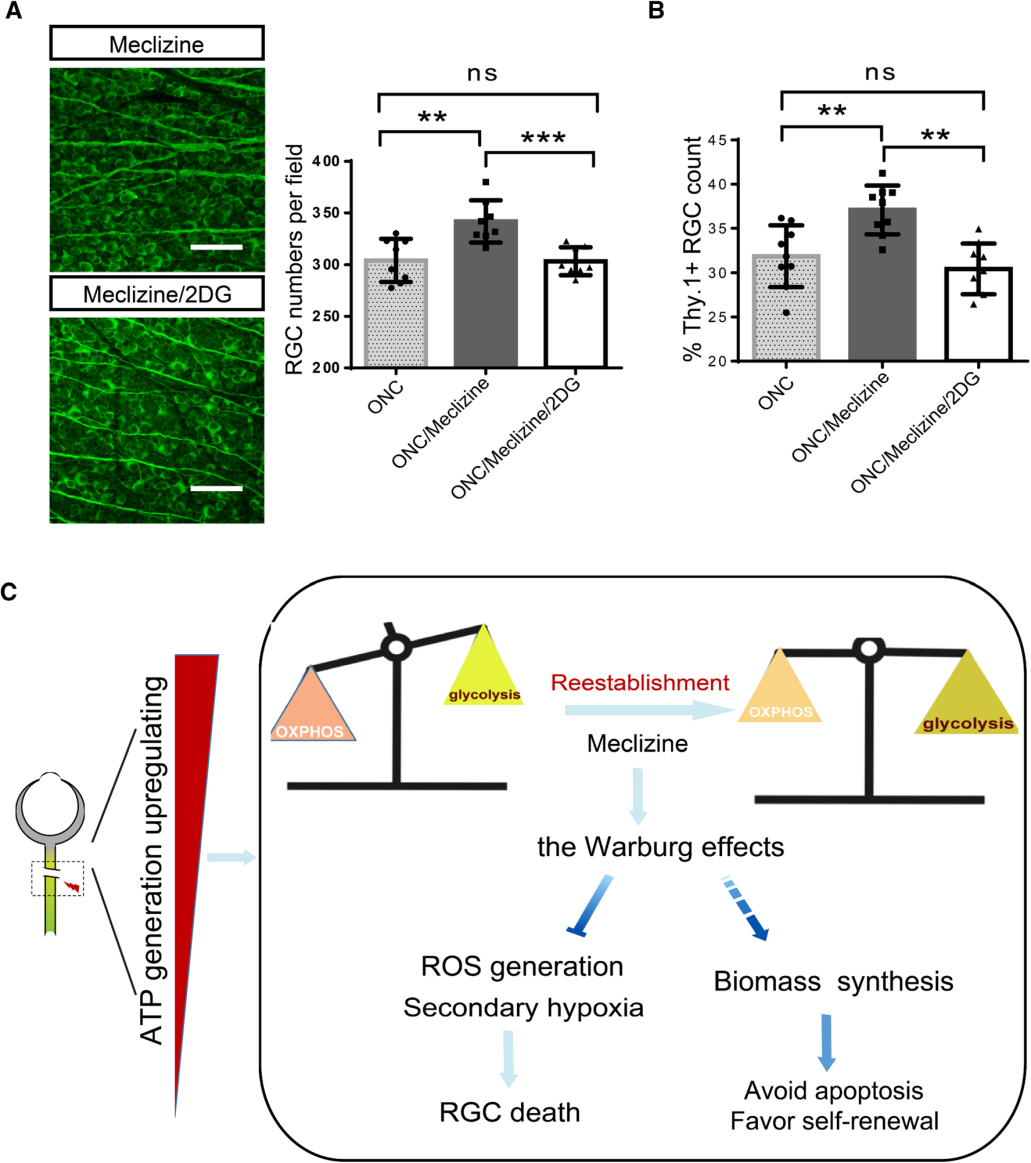
Meclizine pretreatment protects RGCs from death, and this is reversed by 2DG. **A** Meclizine pretreatment prevents RGC death, and 2DG supplementation diminishes this effect. Scale bars, 200 μm. Representative images of whole-mount retina stained for Tuj1 in Meclizine-treated group and Meclizine/2DG-treated group. **B** RGC numbers based on flow cytometry (note that they are consistent with the findings from retinal whole mounts). **C** A working model of metabolic redirection toward glycolysis (Warburg effect) to benefit RGC survival. Disproportionate upregulation of oxidative phosphorylation over glycolysis after ONC may confer several vulnerabilities for RGC survival, including reactive oxygen species (ROS) accumulation and secondary hypoxia. However, re-establishing energy homeostasis by restoring the preference for glycolysis is hypothesized to provide survival advantages to the cells, such as favoring macromolecule biosynthesis and promoting self-renewal.

As the preference for mitochondria-derived ATP production is reduced by activating glycolysis, meclizine achieved a favorable reduction in RGC death (Fig. 8A, B). To confirm that activation of glycolysis is indispensable for this neuroprotective effect, we injected 2DG into the meclizine-protected ONC model and found a mitigation of the positive effect manifested as further RGC loss (Fig. 8A). Therefore, the disproportionate activation of respiration following acute axon injury is a major contributor to RGC damage, and the redirection of energy metabolism toward glycolysis may enhance neuronal survival (Fig. 8B).

## Discussion

To help injured neurons better survive an injury energetically, we must determine their reactions to intense post-injury energy demands [5]. Metabolic plasticity enables cells to adapt to acute energy demands, with which neurons are not typically associated. However, in the present study, the optic nerve and retina displayed metabolic activation 1 day after injury, with signs of increased ATP and ADP levels and enhanced glycolysis and oxidative phosphorylation (Figs 1 and 2). These results differ from the findings of several *in vitro* studies in which neurons tend to display energy deficits after trauma originating from mitochondrial degeneration [7, 46], but they are consistent with an *in vivo* study showing that injury increases the number of motile mitochondria during the early phase of motor nerve regeneration [47]. This discrepancy may be explained by differences between *in vitro* and *in vivo* conditions. *In vivo*, neurons are supported by the surrounding environment, particularly relying on astrocytes to provide lactate and pyruvate for oxidative phosphorylation [48–50]. Energetic support from activated astrocytes to neurons is important during early neurodegeneration and periods of increased energy demand [51–53], while isolated cultured neurons are typically cultured in 25 mmol/L glucose, a hyperglycemic environment that may disturb glucose metabolism, and these neurons rarely survive on their own. The mitochondrial degeneration *in vitro* may reflect ongoing neuronal death with inactive neuron repair and recovery after damage. Notably, we monitored the energy status at the tissue level (whole retina with a mixture of cells and optic nerve with predominantly RGC axons), because it is technically difficult to separate the metabolism of neurons and glia. Thus, the potential neuronal origin of these metabolic changes remains unclear. Measurements at the cellular level are needed to address this problem in future studies.

Although intense energy production is vital for RGC survival after ONC (Fig. 3), increasing the energy level largely failed to promote RGC survival. Our results suggested that inherently high energy levels in the injured optic nerve have no significant effect on RGC survival (Fig. 4A), probably because the spontaneous metabolic activation in the optic nerve and retina is sufficient to satisfy most urgent energy demands during early trauma. Energy-boosting neuroprotection has exhibited great potential in several chronic neurodegenerative conditions, such as Huntington’s disease and Parkinson’s disease [54–56]. However, during acute clinical neuronal injury, such as stroke, this approach is ineffective or even detrimental [57], similar to our acute ONC model. The timing of treatment during the progression of neuronal demise may be a factor. Our results revealed that the increased ATP levels gradually declined over time (1, 3, and 7 days) in the ONC model (Fig. 1F), indicating that the activated energetic response may be an acute adaptation during the early stage of trauma and that energy exhaustion may occur in the later stage of acute neuronal trauma, thus mimicking a chronic neurodegenerative state. Evidence related to glaucoma, a chronic optic nerve injury, also supports our suspicion. RGCs from DBA/2J mice with early-stage glaucoma show higher expression of oxidative phosphorylation genes than those from healthy mice [58]; however, mice with later-stage chronic glaucoma display mitochondrial dysfunction and energy compromise [59], and patients with advanced primary open-angle glaucoma suffer from oxidative phosphorylation complex-I damage and impaired ATP synthesis [60, 61].

We found increased glycolysis and oxidative phosphorylation and that oxidative phosphorylation temporarily outperforms other pathways, providing a stronger response to the increased demand (Fig. 5). The relative contributions of glycolytic flux and oxidative phosphorylation under normal conditions or in response to changing energy requirements in the CNS have not been previously described, potentially because of the lack of available measurement tools and sensitive assays. Our conclusion was first drawn according to calculations using formulas. However, statistical analysis cannot provide an accurate value for the percentage increase or decrease. As more advanced techniques emerge, these proportions should be precisely measured.

Our data showed that prominent activation of oxidative phosphorylation was associated with a poor prognosis for RGC survival (Fig. 6), as suggested by comparisons between PTEN conditional knockout mice and wild-type mice. Meclizine is an ‘older’ FDA-approved compound that has been shown in a drug screen and multiple animal models to be capable of reprogramming energy metabolism [44]. Our study found that, by redirecting metabolism toward glycolysis using meclizine, RGC survival was promoted (Fig. 8A, B). Why is the pharmacological augmentation of glycolysis protective? The underlying causes may be similar in our model and in the “Warburg effect” in tumors (Fig. 8) [62–64]. Overactive oxidative phosphorylation can cause extensive ROS production, and the resultant oxidative stress has been confirmed to be a major cause of RGC apoptosis after axon injury [65–67]. In addition, respiratory substrate influx into mitochondria may cause sensitivity to mitochondria-mediated apoptosis [68–70], which may also lead to excessive respiration-induced RGC loss in the ONC model. Furthermore, a metabolic preference for glycolysis is an inherent trait of neural progenitor cells [71, 72], which may indicate neural stem cell activation in our model, potentially representing an additional step toward neuronal protection and regeneration. In conclusion, we provided clues that pathologically augmented mitochondrial respiration may be a double-edged sword in the injured optic nerve. That is, excessive activation of mitochondrial respiration causes massive secondary damage to RGCs despite producing energy (Fig. 8C). This model can also explain why increased internal energy generation is not significantly correlated with RGC survival, as illustrated in Fig. 3. Exactly how these imbalances in the reallocation of energy production pathways lead to neuropathology warrants further investigation. Overall, as a first step toward elucidating how neuronal energy metabolism adapts to axonal insults *in vivo*, our study puts forward a potential bioenergetic strategy to facilitate neuronal survival in the traumatized CNS that involves shifting energy metabolism toward glycolysis (the Warburg effect).

## Electronic supplementary material

Below is the link to the electronic supplementary material.

Supplementary material 1 (PDF 70 kb)
