# Supplementary material for: Altered Energy Metabolism During Early Optic Nerve Crush Injury: Implications of Warburg-Like Aerobic Glycolysis in Facilitating Retinal Ganglion Cell Survival
Source: Neurosci Bull. 2020 Apr 10;36(7):761–77. doi: 10.1007/s12264-020-00490-x (PMC7340706; doi:10.1007/s12264-020-00490-x)
Supplement: Supplementary file 1 — Supplementary material 1 (PDF 70 kb) [file 12264_2020_490_MOESM1_ESM.pdf]

Table S1. Qualitative analysis of LDH histochemistry as in Figure 2E.

Group	High Positive<br>+++ (%)	Positive<br>++ (%)	Low Positive<br>+ (%)	Negative<br>- (%)	Overall Rating
CON	0.1032	9.3041	34.4133	56.1794	Low Positive
ONC	10.963	65.097	23.58	0.045	Positive

Quantitative analysis was conducted with ImageJ IHC Profiler. Histochemical staining of LDH in optic nerves was automatically categorized as high positive/positive/low positive/negative and then counted and rated overall by the software. LDH, lactate dehydrogenase; CON, control; ONC, optic nerve crush.

Table S2. Qualitative analysis of LDH histochemistry as in Figure 7C.

Group	High Positive<br>+++ (%)	Positive<br>++ (%)	Low Positive<br>+ (%)	Negative<br>- (%)	Overall Rating
ONC	13.05	58.58	27.41	0.96	Positive
ONC/Meclizine	72.47	23.87	3.11	0.55	High Positive
CON	0.14	14.01	35.68	50.27	Low Positive
Meclizine	34.20	31.65	33.14	1.00	Positive

ONC, optic nerve crush; ONC/Meclizine, optic nerve crush with Meclizine pretreatment; CON, control; Meclizine, with Meclizine treatment.
